# Environmental Contamination by Dog’s Faeces: A Public Health Problem?

**DOI:** 10.3390/ijerph10010072

**Published:** 2012-12-24

**Authors:** Vittoria Cinquepalmi, Rosa Monno, Luciana Fumarola, Gianpiero Ventrella, Carla Calia, Maria Fiorella Greco, Danila de Vito, Leonardo Soleo

**Affiliations:** 1 Department of Interdisciplinary Medicine, Occupational Medicine Section , University of Bari, Piazza Giulio Cesare, 11, 70124 Bari, Italy; E-Mails: 5vittoria84@libero.it (V.C.); l.soleo@medlav.uniba.it (L.S.); 2 Department of Basic Medical Sciences, University of Bari, Piazza Giulio Cesare, 11, 70124 Bari, Italy; E-Mails: lfumarola@midim.uniba.it (L.F.); carla.calia@virgilio.it (C.C.); d.devito@igiene-seconda.uniba.it (D.V.); 3 Department of Veterinary Public Health, University of Bari, Valenzano, 70010 Bari, Italy; E-Mails: gianpieroventrella85@libero.it (G.V.); grecofiorella@virgilio.it (M.F.G.)

**Keywords:** dogs, resistant strains, public health, fecal sample, zoonosis, bacteria

## Abstract

The risk to public health from the large number of dog stools present on streets of urban areas is cause for concern. Dog faeces may be a serious hazard because they may contain microorganisms that are both pathogenic to humans and resistant to several classes of antibiotics. The aim of this study was to evaluate the potential for zoonotic infections and for the presence of antibiotic resistant bacteria in canine faeces which contaminates the urban environment. A total of 418 canine faecal samples were collected from streets in seven areas of Bari, Southern Italy. We have isolated multi-drug resistant *Enterococci* and meticillin-resistant *Staphylococcus aureus* from these dog faecal samples. The presence of the resistant bacteria in an urban environment may represent a public health hazard which requires control measures by competent authorities. No *Salmonella*, *Yersinia *or *Campylobacter* species were isolated. *Giardia *cysts were detected in 1.9% of the samples. The predominant *Enterococcus* species were *E.faecium* (61.6%), *E. gallinarum* (23.3%) and *E. casseliflavus* (5.5%). Other species, including *E*. *faecalis* were also isolated. These strains were resistant to clindamycin (86.3%), tetracycline (65.7%), erythromycin (60.27%) and ampicillin (47.9%). High-level aminoglycoside resistance (HLAR) was found in 65.7% of *enterococci*. Resistance to three or more antibiotics and six or more antibiotics were observed in 67.12% and 38.4% of *Enterococcus *spp., respectively. Resistance to vancomycin and teicoplanin was not detected in any of the *Enterococcus* spp. isolated. Methicillin-resistant *Staphylococcus aureus* was isolated in 0.7% of the faecal samples. Canine faeces left on the streets may represent a risk factor for transmission of microorganisms and a reservoir of multidrug- resistant bacteria thus contributing to the spread of resistance genes into an urban area.

## 1. Introduction

Dogs and cats live in close contact with humans. In particular, dog numbers have increased in industrialised countries. The presence of dog faeces in urban settings due to the habit of dog owners of not removing dog faeces from the street may represent a problem for hygiene and public health. Dog faeces may contain several types of microorganisms potentially pathogenic for humans. Bacteria that are pathogens for the intestinal tract and cause diarrhoea include *Campylobacter*, *Salmonella*, *Yersinia *and *E. coli* [1,2,3,4]. Dog faeces may also contribute to the diffusion of protozoa such as *Giardia* and *Cryptosporidium* [5] and of roundworms such as *Toxocara canis * [6]. Recently, there has been increased evidence that pets and their stools may be a reservoir for antibiotic-resistant bacteria [7], posing a new threat to public health. In particular, the presence of vancomycin-resistant *enterococci* (VRE) in pet animals, including dogs, has been reported [8]. A relatively high occurrence (7–23%) of VRE, mainly *E. faecium* in dogs living in urban areas has also been reported in Europe [9,10,11]. Furthermore, *enterococci* with high-level aminoglycoside resistance (HLAR) have been described in strains isolated from both humans and animals [12]. In addition, methicillin-resistant *Staphylococcus aureus* (MRSA) has been found in the stools of dogs and has been isolated from both infected and colonised pet animals [13,14,15,16,17,18,19]. The MRSA isolated from pet animals resembles MRSA strains isolated in a hospital setting suggesting transmission of these strains from animals to human or *vice versa* [15]. Of additional concern is also the decription of the isolation of *E. coli* producing extended-spectrum beta-lactamases from dogs [20]. Thus, dogs represent a potential source of antimicrobial—resistant microorganisms, especially considering the overuse of antimicrobials in companion animals. In 1997, the number of cats and dogs was estimated to be above 70 million in European countries [21] and, today, close physical contact with dogs is more frequent because household pets are considered family members. The aim of this study was to evaluate the presence of diverse microorganisms responsible for transmittable zoonoses in dog faecal samples collected from different streets within the city of Bari. In addition, because few studies have dealt with antimicrobial resistance in bacteria isolated from dog stools, the presence of VRE, HLAR *enterococci* and MRSA was also assessed. 

## 2. Materials and Methods

From February to September 2010, a total of 418 dog faecal samples (44% of fecal samples were fresh feces and 56% were aged feces) were collected from sidewalks and streets of main roads of seven sub-areas of the city of Bari, with approximately 60 different samples from each subarea. A large quantity of each formed stool specimen was collected with a plastic spoon, placed in faecal plastic containers and sent to the laboratory of Microbiology of the Department of Basic Medical Sciences of the Faculty of Medicine, University of Bari, Italy and processed within four hours.

### 2.1. Bacteriological Investigations

The isolation of *Salmonella* was performed by first transferring a large amount of faecal sample into 10 mL of Selenite broth (Oxoid, Milan, Italy). After incubation at 37 °C for 18 h, 0.1 mL of the selective broth was inoculated onto *Salmonella Shigella* agar (Oxoid) and incubated at 37 °C for 24–48 h. Colonies showing typical *Salmonella* morphology (lactose-negative, with a black centre) were struck on Triple Sugar Iron (TSI) slant (Oxoid). When *Salmonella* was suspected, biochemical identification was performed by an automated system (MicroScan WalkAway, Siemens, Milan, Italy). 

For *Campylobacter* isolation, faecal samples were directly inoculated onto Skirrow medium plates (Oxoid), and incubated at 42 °C for 48–72 h under a microaerophilic atmosphere (gas generating kit, Oxoid). 

For *Yersinia* isolation, aliquots of faecal samples were inoculated into CIN agar (Oxoid). Suspected colonies appearing as a bull’s eye were struck on TSI slant. When *Yersinia * was suspected the urease test was performed, and, if positive, biochemical identification was performed by the above-mentioned automated system.

For isolation of *Enterococcus* spp., aliquots (1 g) of stools were inoculated in 5 mL of Enterococcosel Broth (Becton Dickinson, Milan, Italy) and, after incubation for 18 h at 37 °C, aliquots were struck on Enterococcosel Agar (Becton Dickinson) and incubated for 24–48 h. Suspected colonies were identified as *Enterococcus *spp. by the above-mentioned automated system. 

For the isolation of MRSA, aliquots of stools were inoculated in Mueller-Hinton broth with the addition of 6.5% NaCl. After incubation for 18–24 h at 37 °C, aliquots were struck onto mannitol salt agar supplemented with oxacillin (2 μg/mL) and incubated for 24 h at 37 °C. *S. aureus* was identified via a positive latex agglutination test (Slidex Staph-Plus, bioMérieux, Florence, Italy) and the automated system. Confirmation of methicillin resistance was carried out by growth on Mueller-Hinton agar supplemented with NaCl (4% w/v) and with 6 μg/mL of oxacillin and followed by polymerase chain reaction (PCR) detection of the mecA gene as described below.

### 2.2. Susceptibility Testing

Susceptibility to vancomycin, teicoplanin, ampicillin, erytromycin, tetracycline, levofloxacin, penicillin, piperacillin-tazobactam, trimethoprim-sulfamethoxazole, chloramphenicol, clindamycin and amoxicillin/clavulanic acid was determined by the disk diffusion method as described in Clinical and Laboratory Standards Institute (CLSI) guidelines [22]. Mueller Hinton agar and antimicrobial impregnated disks (Biolife, Milan, Italy) were used.

Detection of high-level aminoglycoside resistance in *enterococci* was performed according to the method previously described [23] by inoculating a 10 μL suspension of *Enterococcus *spp. equivalent to a 0.5 Mc Farland Standard onto Brain Heart Infusion (BHI) agar supplemented with 500 μg of gentamicin per mL and BHI supplemented with 2,000 μg of streptomycin per mL. The presence of more than one colony or a haze of growth was read as resistant.

### 2.3. Molecular Studies

The *vanA*, *vanB*, *and vanC *genes were targeted with specific primers in separate PCRs according to Dukta- Malen *et al.* [24]. As positive controls, *E. faecium* ATCC 51559 was used for *vanA*, *E. faecalis *ATCC 51299 was usedfor *vanB*, and *E. gallinarum* ATCC 49573 and *E. casseliflavus* ATCC 25788 were used for *vanC_2_/C_3_*. The presence of the *mecA* gene in *S. aureus* was evaluated as previously described [25]. A molecular diagnostic identification for *Staphylococcus pseudintermedius *was performed using the primers pse-F_2 _and pse-R_5 _according to Sasaki *et al.* [26].

### 2.4. Giardia Detection

All 418 dog faecal samples were evaluated for the presence of *Giardia* by microscopic examination after the use of the sedimentation flotation technique [27]. In addition, 190 of the 418 samples were analysed by using a commercially available EIA for *Giardialamblia* antigen (Serazym^®^ *Giardia lamblia *ELISA kit, DID, Milano, Italy) which detect *Giardia lamblia* using labelled polyclonal antibodies. After the kit was no longer commercially available from the manufacturers, 25 further samples were analyzed by using an Immunochromatographic test (Giardia Dipstick, DID), which is a qualitative lateral flow immunoassay for the detection of *Giardia* antigen in faeces samples. Both assays were performed according to the manufacturers’ instructions.

## 3. Results

All of the 418 dog stool samples examined were negative for the presence of *Salmonella*, *Campylobacter* or *Yersinia*. *Giardia *spp. was found in only one of the 418 samples analysed by microscopy. When the same samples were analysed by an ELISA (190 samples) or the immunochromatographic method (25 samples), only four (1.9%) were positive. The positive result was confirmed by repeating the exam twice. Sixty-eight of 418 faecal samples were positive for *enterococci* and 73 strains were isolated two different enterococcal species were identified in one faecal sample and three in another sample). The isolates were identified as *E. faecium* (45/73, 61.6%), *E. gallinarum* (17/73, 23.3%) and *E. casseliflavus* (4/73, 5.5%). Other species isolated (*E. raffinosus*, *E. avium *and *E. durans*) accounted for 0.027% of the samples. *E. faecalis *was identified only in one specimen.

The pattern of antibiotic resistance in *Enterococcus spp*. was analysed, and Table 1 shows the results obtained for each species of *Enterococcus *isolated. *Enterococcus* spp. were resistant to clindamycin (86.3%), tetracycline (65.7%), erythromycin (60.27%), ampicillin (47.9%), penicillin (46.6%), piperacillin-tazobactam (43.8%), amoxicillin-clavulanic acid (34.2%), levofloxacin (23.3%) and trimethoprim-sulfametoxazole (9.6%) while a low percentage of resistance to cloramphenicol (1.4%) was found.

**Table 1:** Antimicrobial resistance of *enterococci* isolated from faecal samples of dogs detected by the disk diffusion method.

Antibiotic No. resistant (% resistant)	No. ( % resistant )						
	*E. faecium* (n = 45)	*E. gallinarum* * (n = 17)	*E. casseliflavus* * (n = 4)	*E. raffinosus* (n = 2)	*E. faecalis* (n = 1)	*E. avium *(n = 2)	*E. durans/hirae* (n = 2)
Clindamycin n = 63 (86.3%)	38 (84.4)	15 (88.23)	4 (100)	2 (100)	1 (100)	2 (100)	1 (50)
Tetracycline n = 48 (65.7%)	33 (73.3)	10 (58.9)	2 (50)	2 (100)	1 (100)	0 (0)	0 (0)
Erythromycin n = 44 (60.27%)	35 (77.7)	4 (23.52)	2 (50)	1 (50)	1 (100)	0 (0)	1 (50)
Ampicillin n = 35 (47.9%)	30 (66.6)	2 (11.8)	0 (0)	0 (0)	1 (100)	0 (0)	2 (100)
Penicillin n = 34 (46.6%)	33 (73.3)	0 (0)	0 (0)	0 (0)	0 (0)	0 (0)	1 (50)
Piperacillin-Tazobactam n = 32 (43.8%)	30 (66.6)	1 (5.9)	0 (0)	0 (0)	0 (0)	0 (0)	1 (50)
Amoxicillin + clavulanic acid n = 25 (34.2%)	23 (51.1)	1 (5.9)	0 (0)	0 (0)	0 (0)	0 (0)	1 (50)
Levofloxacin n = 17 (23.3%)	15 (33.3)	1 (5.9)	0 (0)	0 (0)	0 (0)	0 (0)	1 (50)
Trimethoprim-Sulfamethoxazole n = 7 (9.6%)	3 (6.6)	2 (11.8)	0 (0)	0 (0)	0 (0)	0 (0)	2 (100)
Chloramphenicol n = 1 (1.4%)	0 (0)	1 (5.9)	0 (0)	0 (0)	0 (0)	0 (0)	0 (0)

* These species was susceptible by the disk diffusion method but showed a MIC of 6–12 mg/L when tested by the E-test.

**Table 2:** Multiple antimicrobial resistances among *enterococci* isolated from faecal samples of dogs.

Species		No. Resistant (%)									
	No. Antimicrobials	0	1	2	3	4	5	6	7	8	9
*E. faecium* (n = 45)		1 (2.2 )	3 (6.7)	3 (6.7 )	4 (8.9)	1 (2.2 )	7 (15.5)	6 (13.3 )	10 (22.2)	9 (20)	1 (2.2)
*E. gallinarum* (n = 17)		0 (0)	3 (17.6)	9 (52.9)	2 (11.8)	1 (5.9)	1 (5.9)	0 (0)	1 (5.9)	0 (0)	0 (0)
*E. casseliflavus* (n = 4)		0 (0)	0 (0)	2 (50)	0 (0)	50 (2)	0 (0)	0 (0)	0 (0)	0 (0)	0 (0)
*E. faecalis* (n = 1)		0 (0)	0 (0)	0 (0)	0 (0)	100 (1)	0 (0)	0 (0)	0 (0)	0 (0)	0 (0)
*E. avium* (n = 2)		0 (0)	0 (0)	2 (100 )	0 (0)	0 (0)	0 (0)	0 (0)	0 (0)	0 (0)	0 (0)
*E. raffinosus *(n = 2)		0 (0)	0 (0)	1 (50)	1 (50)	0 (0)	0 (0)	0 (0)	0 (0)	0 (0)	0 (0)
*E. durans/hirae *(n = 2)		0 (0)	0 (0)	0 (0)	1 (50)	0 (0)	0 (0)	0 (0)	0 (0)	0 (0)	1 (50)
Total (n = 73)		1 (1.4)	6 (8.2)	17 (23.3)	8 (10.9)	5 (6.8)	8 (10.9)	6 (8.2)	11 (15.1 )	9 (12.31)	2 (2.7)

Although all strains of *E. casseliflavus* and *E. gallinarum *appeared susceptible to vancomycin by the disk diffusion test, vancomycin minimum inhibitory concentration was present and ranged between 6 and 12 mg/L, when tested by the E-test. This is indicative of low resistance to vancomycin and, as reported in the literature, not detectable by the disk diffusion method [28]. MIC to teicoplanin of these species ranged between 0.25-0.5 mg/L. All 17 strains of *E. gallinarum* possessed the *vanC*_1_gene and lacked the *van C_2_/C_3_* gene. In all four strains of *E. casseliflavus* the van C_2_/C_3_ gene was detected. Multiresistance patterns, defined as resistance to 3 or more antibiotics was observed in 49 of 73 strains of *Enterococcus* (67.12%). Resistance to 6 or more antibiotics was found in 38.4% of strains (Table 2). 

**Figure 1:**
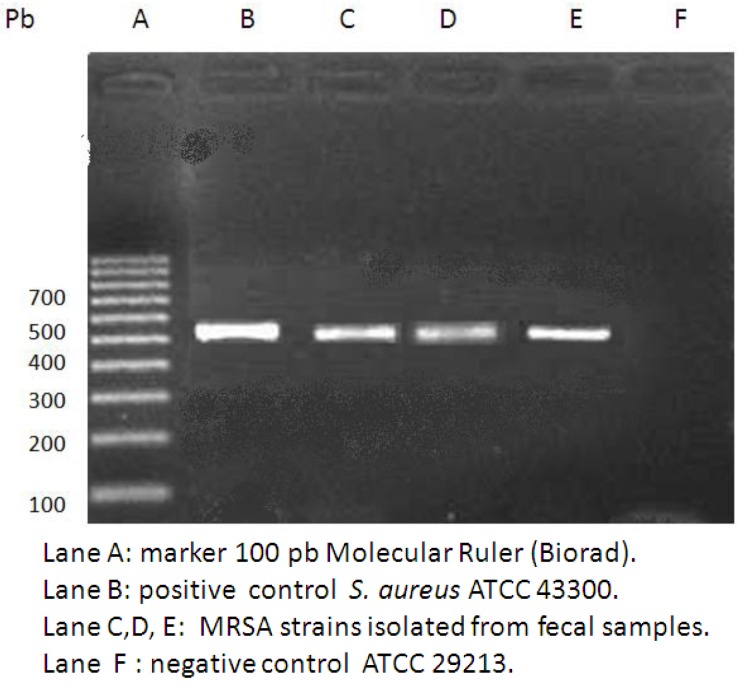
Detection of the *mecA *gene in 3 strains of *S. aureus* isolated from faecal samples.

**Figure 2:**
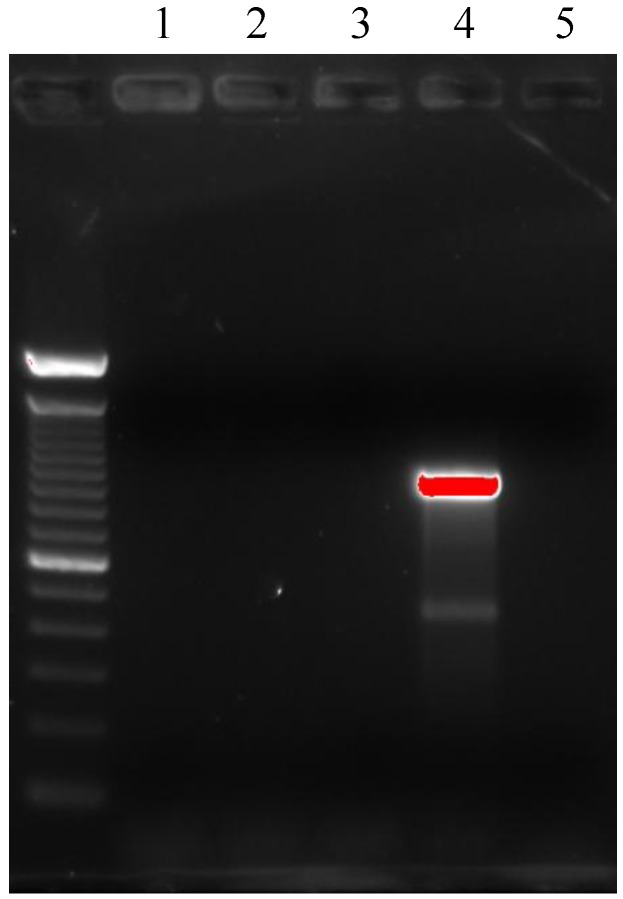
Electrophoresis after PCR for *S. pseudintermedius* identification on a 1.0% agarose gel.Lane 1: molecular marker: 100 bp DNA ladder TrackLT (Invitrogen, Monza, Italy); Lanes 2-4: samples; Lane 5: positive control, canine isolate *S. pseudintermedius* (926 bp band).

**Figure 3:**
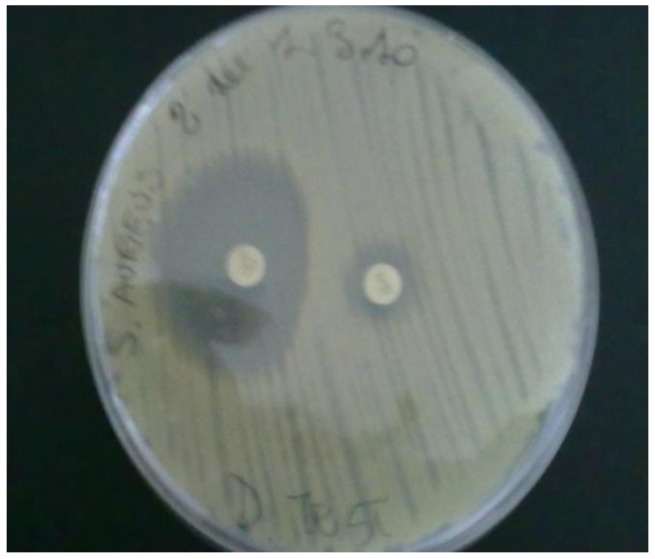
D test performed on *S. aureus* isolated from dogs for the detection of inducible clindamycin resistance. Staphylococcal isolates showing resistance to erythromycin while being sensitive to clindamycin and giving D shaped zone of inhibition around clindamycin with flattening towards erythromycin disc were reported as resistant to clindamycin.

High-level gentamicin resistance (MIC ≥ 500 mg/L) and/or high-level streptomycin resistance (MIC ≥ 1000 mg/L) were detected in 65.7% of *Enterococcus spp*. In particular 82.2% (37/45) of the *Enterococcus faecium*, 50% (2/4) of the *E. cassseliflavus*, 17.6% (3/17) of the *E. gallinarum*, 50% (1/2) of the *E*. *durans*, 100% (1/1) of the *E. faecalis *and 100% (2/2) of the *E. avium* and *E. raffinosus* were found to be HLAR (Table 3). Only 3 strains of methicillin-resistant *S. aureus* (MRSA) were isolated. This resistance was demonstrated both by phenotypic and molecular (PCR positivity for mecA gene) methods (Figure 1). All of these strains were not *S. pseudintermedius* as demonstrated by molecular methods (Figure 2). All these strains were resistant to erythromycin, tetracycline and susceptible to trimethoprim-sulphamethoxazole and cloramphenicol. Resistance to amikacin and gentamicin was detected in two of these strains. One strain was resistant to both levofloxacin and ciprofloxacin. Inducible clindamycin resistance as detected by the Double Disk Diffusion test (D-test) was present in all three MRSA strains (Figure 3).

**Table 3:** High-level aminoglycoside resistance (HLAR) in *enterococci*.

	No. Resistant (%)							
	*E. faecium*	*E. gallinarum*	*E. casseliflavus*	*E. raffinosus*	*E. faecalis*	*E. avium*	*E. durans*	Total
**Gentamicin only**	10/45 (22.2)	0/17 (0)	0/4 (0)	0/2 (0)	0/1 (0)	1/2 (50)	0/2 (0)	11/73 (15.1)
**Streptomycin only **	1/45 (2.2)	1/17 (5.8)	1/4 (25)	2/2 (100)	1/1 (100)	1/2 (50)	0/2 (0)	7/73 (9.6)
**Gentamicin** ** +** ** Streptomycin**	26/45 (57.8)	2/17 (11.8)	1/4 (25)	0/2 (0)	0/1 (0)	0/2 (0)	1/2 (50)	30/73 (41.1)
**Total HLAR**	37/45 (82.2)	3/17 (17.6)	2/4 (50)	2/2(100)	1/1 (100)	2/2 (100)	1/2 (50)	48/73 (65.7)

## 4. Discussion

Dog faeces in urban settings may represent an important source of microorganisms potentially pathogenic for both dog owners and the community. In our study we did not isolate strains of *Salmonella* and this may be because only dogs consuming contaminated raw meat can shed *Salmonella* in their faeces, although we have no information about diets in these dogs [29,30]. Our results are in agreement with those of Tarsitano *et al.*, who in a recent study conducted in Bari, Southern Italy, were also unable to identify these bacteria in faecal samples even when a PCR assay specific for the *inv*A gene of *Salmonella *was used [31]. 

The absence of *Campylobacter *spp. may also be due to loss of viability of *Campylobacter* in the environment. In addition, very young dogs are more often infected with *Campylobacter* species than older dogs and therefore may not constitute a health hazard to the public [32]. Because we cannot know the age of dogs, we can only hypothesise that the fecal samples analysed in our study were not from very young dogs. Furthermore, the medium used in our study contains antimicrobial agents that are known to be inhibitory for other *Campylobacter* species, including *C. lari*, *C. sputorum*, *C. upsaliensis*, *C. fetus* and *C. hyointestinalis* [32]. Thus, we cannot exclude the possibility that these other *Campylobacter *spp. could have been present in the faecal samples. Additionally, *Yersinia *spp. were not found.

The absence of all *Salmonella*, *Yersinia*, and *Campylobacter *sp. is not related to previous immunization as vaccines for these enteropathogens are not used in Italy. 

We have identified *Giardia* antigen in 1.9% of the examined samples by use of an ELISA. *Giardia *is a protozoan parasite with worldwide distribution that infects human as well as pet animals including dogs. Cysts are the infective stage and can persist in the environment. A prevalence of 19-21.3% *Giardia* spp. in dogs has been reported in Italy [5,33]. In two separate studies examining canine faeces left on the streets of urban areas, *Giardia * spp. was detected in 7.7% and 30.8% of cases [34,35]. Because of the low sensitivity of microscopic detection of *Giardia*, its presence in our samples may have been underestimated. We observed embryonated eggs of *Toxocara canis* only in one sample, a level of occurrence similar to that reported in another Italian studies [34]. 

Few studies have examined healthy dogs for the presence of antibiotic-resistant *enterococci*, and therefore, the extent of antimicrobial resistance in companion animals is poorly understood [36]. In previous studies, up to 37% of rectal samples of dogs examined were positive for *enterococci*, with the species most frequently isolated being *E. faecalis* and *E. hirae* [37,38].*Enterococci* can colonise the intestinal tract of humans, *E. faecalis* being responsible for 80-90% of human enterococcal infections, whereas *E. faecium *represents the next most common causative agent [39].

*Enterococci* are intrinsically resistant to several classes of antibiotics. High rates of resistance to several antibiotics used in humans, including erythromycin and tetracycline, have been reported in *Enterococcus *spp. isolated from dogs in Italy [40]. Resistance to vancomycin, especially in the hospital settings, has emerged in *Enterococcus *spp. [41]. Dogs represent potential sources for the spread of antimicrobial resistance due to the extensive use of antimicrobial agents in these animals and their close physical contact with family members [42]. The ability of antibiotic-resistant *enterococci* isolated from non-human sources (such as raw meat, animal faeces, sewage) to colonise human or their ability to transfer resistance to human *enterococci* is not well known. Some studies have failed to demonstrate a relationship between antibiotic-resistant (including glycopeptides) *enterococci* isolated from human and those isolated from non-human sources [43,44], while other studies have reported a genetic relationship between *Enterococcus *spp. isolated from humans and those isolated from animals, including dogs [11].

HLAR resistance in *Enterococcus *spp. is related to the presence of a gene encoding aminoglycoside -modifying enzymes [45]. In our study 65.7% of *Enterococcus *spp. isolated from dog faeces exhibited a high level of aminoglycoside resistance. In a previous study we have observed that 33.2% of *E. faecalis* and 31.3% of *E. faecium* isolated from patients admitted to Policlinico Hospital, Bari, Southern Italy were found to be HLAR [46].

High-level resistance to aminoglycoside poses serious problems for combined penicillin and gentamicin therapy due to the reduction of the synergy between these two antibiotics. The incidence of HLAR in *Enterococcus *spp. isolated from dogs is of concern, considering that this resistance is usually mediated by conjugative and non-conjugative plasmids and that gene exchange may occur also between *Enterococcus* spp. and *Staphylococcus spp*. Our data suggest that dog faecal samples could contribute to the spread of HLAR *enterococci* and it should be a matter of concern for public health authorities. We need infection control measures to help prevent the diffusion of antibiotic resistance in the urban areas [47]. 

We isolated MRSA from 0.7 % of the samples, comparable to other studies where MRSA has been isolated from 0.4 % to about 3% of dogs [48,49]. MRSA isolated from pets (dogs and cats) also closely resembled isolates disseminated in hospitals [48]. Dogs, therefore, may represent a public health burden in disseminating MRSA outside of hospitals [49,50,51,52,53]. Transmission of bacteria between dogs or other companion animals and humans may occur by direct contact or through the domestic environment. Children are particularly at risk because of their direct contact with the floor and contaminated carpets. We cannot exclude the possibility that we have underestimated the number of bacteria, including the number of potentially infectious bacteria, especially when “aged” samples were analysed. This would mean that our results are even more interesting and crucial from the public health standpoint.

In conclusion, bacteria pathogenic to humans were not found in the faecal samples of dogs collected from public streets of Bari, whereas *Giardia *was present in the 1.9% of samples. Multidrug-resistant *Enterococcus *spp., including HLAR, and MRSA were isolated from dog faecal samples present in the urban environment, confirming that the environmental contamination of the public streets of Bari may be a reservoir of resistant bacteria thus represents a new problem for public health.

## 5. Conclusions

There are few studies dealing with the presence of microorganisms pathogenic to humans in dog faeces and that address the role of dog faeces in urban areas as reservoir of multidru-resistant (MDR) bacteria such as *Enterococcus *and *S. aureus*. Our study has demonstrated that although no pathogenic bacteria for humans were present in this study in the city of Bari, Italy, the presence of *Giardia *spp. were found together with MDR *Enterococcus *spp. and methicillin-resistant *S. aureus*. Because genes encoding resistance to antibiotics may be exchanged between bacteria and because contact between pets and their owners is closer than in the past, our study suggests that the contamination of urban streets with dog faeces containing MDR microorganisms represents a problem for public and environmental health.
